# Efficacy and safety of 3D-printed artificial vertebral bodies for spinal tumor resection and reconstruction: a systematic review and meta-analysis

**DOI:** 10.1186/s13018-026-06788-2

**Published:** 2026-04-29

**Authors:** Qiaojuan Wang, Jiyao Chen, Shutao Zheng, Jun Ma

**Affiliations:** 1 Department of pathology, School of basic medicine, Xinjiang Medical University, Xinjiang Uygur Autonomous Region, Urumqi, 830017 People’s Republic of China; 2 Department of pediatric orthopedics, The First Affiliated Hospital of Xinjiang Medical University, Xinjiang Uygur Autonomous Region, Urumqi, 830011 People’s Republic of China; 3 State Key Laboratory of Pathogenesis, Prevention, Treatment of Central Asian High Incidence Diseases, Clinical Medical Research Institute, First Affiliated Hospital of Xinjiang Medical University, Xinjiang Uygur Autonomous Region, Urumqi, 830011 People’s Republic of China

**Keywords:** Spinal tumor, 3D printed, Artificial vertebral body, Total en bloc spondylectomy, Meta-analysis

## Abstract

**Objective:**

To investigate the efficacy and safety of 3D printed artificial vertebral body in spinal reconstruction after total en bloc spondylectomy (TES) for spinal tumors.

**Methods:**

We searched PubMed, Embase, ScienceDirect, Web of Science, Cochrane Library, China National Knowledge Infrastructure (CNKI), Wan Fang Database, and Wei Pu Database by computer to collect controlled clinical studies on the efficacy and safety of 3D printed artificial vertebral body and titanium mesh cages (TMCs) in the treatment of spinal tumors after total en bloc spondylectomy (TES) from database establishment to February 2024.

**Results:**

A total of 10 cohort studies and 2 randomized controlled study with a total of 489 patients were included in this study, including 234 patients in the 3D group and 255 patients in the TMC group. The 3D group had lower intraoperative blood loss than the TMC group [mean difference (MD) = − 1.47, 95% CI (− 2.38, -0.55), *P* = 0.002] and operation time was shorter than that of the TMC group [MD = -0.65, 95% CI (− 1.23, − 0.07), *P* = 0.03]. Early postoperative JOA scores improved more significantly in the TMC group [MD = 0.50, 95% CI (0.18, 0.82), *P* = 0.002].

**Conclusion:**

The 3D printing group demonstrated advantages in early postoperative pain relief (VAS) and spinal sequence maintenance (loss of Cobb angle), while there were no statistically significant differences between the two groups in Frankel grading and neurological improvement rates. Notably, the traditional titanium mesh group showed more significant improvements in early postoperative JOA scores.

**Supplementary Information:**

The online version contains supplementary material available at 10.1186/s13018-026-06788-2.

## Introduction

Spinal tumors are divided into primary tumors and metastatic tumors, of which metastatic tumors account for 80% of spinal tumors. The incidence of primary spinal tumors is about 0.4%, of which malignant tumors account for 36% of primary spinal tumors. It has been reported in the literature that 10–30% of patients with primary malignancies will develop spinal metastases in the late stages [1]. Intractable pain, spinal instability, and nerve compression are the most common symptoms, whether primary malignant tumors of the spine or metastatic tumors. 30% of patients with spinal metastases will have a serious impact on quality of life due to pain or nerve compression symptoms [2], and patients with primary spinal malignancies will experience paraplegia and even shorten survival at a later stage [3].

The main goals of surgical treatment of spinal tumors are to remove the tumor, relieve nerve compression and reconstruct the stability of the spine. For patients with primary spinal malignancies and metastases with long survival, total en bloc spondylectomy (TES) can remove the tumor as a whole, thereby achieving a safe tumor-free boundary and achieving the purpose of reducing recurrence. This technique has been shown to be effective in local tumor control and improves survival in patients with spinal tumors [4–6]. However, en bloc resection is invasive, removes the bony structure of the spine, facet joints, ligaments, intervertebral discs, etc., and completely destabilizes the spine and requires reconstruction of spinal continuity and stability. The traditional reconstruction materials include autologous iliac bone block and titanium mesh, but there are some problems such as low fusion rate, collapse and displacement [7].

With the development of 3D printing technology, the application range of 3D printing implants in orthopedics continues to expand. 3D printed artificial vertebral bodies have similar elastic modulus, good histocompatibility and mechanical strength, and microporous structures suitable for bone ingrowth as the human body’s own vertebral bodies, which make it possible to individualize artificial vertebral bodies to reconstruct bone defects after spinal tumor surgery [8]. At present, there are few reports on the comparative study of the stability of titanium mesh and 3D printed artificial vertebral body reconstruction after en bloc resection of spinal tumors, and its clinical application time is short, the sample size of clinical studies is small, and there is a lack of evidence-based medical evidence for the efficacy and safety. In this study, a meta-analysis of the relevant literature was reported as follows.

## Methods

This meta-analysis followed the Cochrane handbook for conducting and the Preferred Reporting Items for Systematic Reviews and Meta-Analysis (PRISMA) guidelines for reporting [9, 10]. Two authors separately conducted literature retrieval, study eligibility, data extraction, and quality assessment with inconsistency solved by discussion and decided by the corresponding author.

### Literature search

**Fig. 1:**
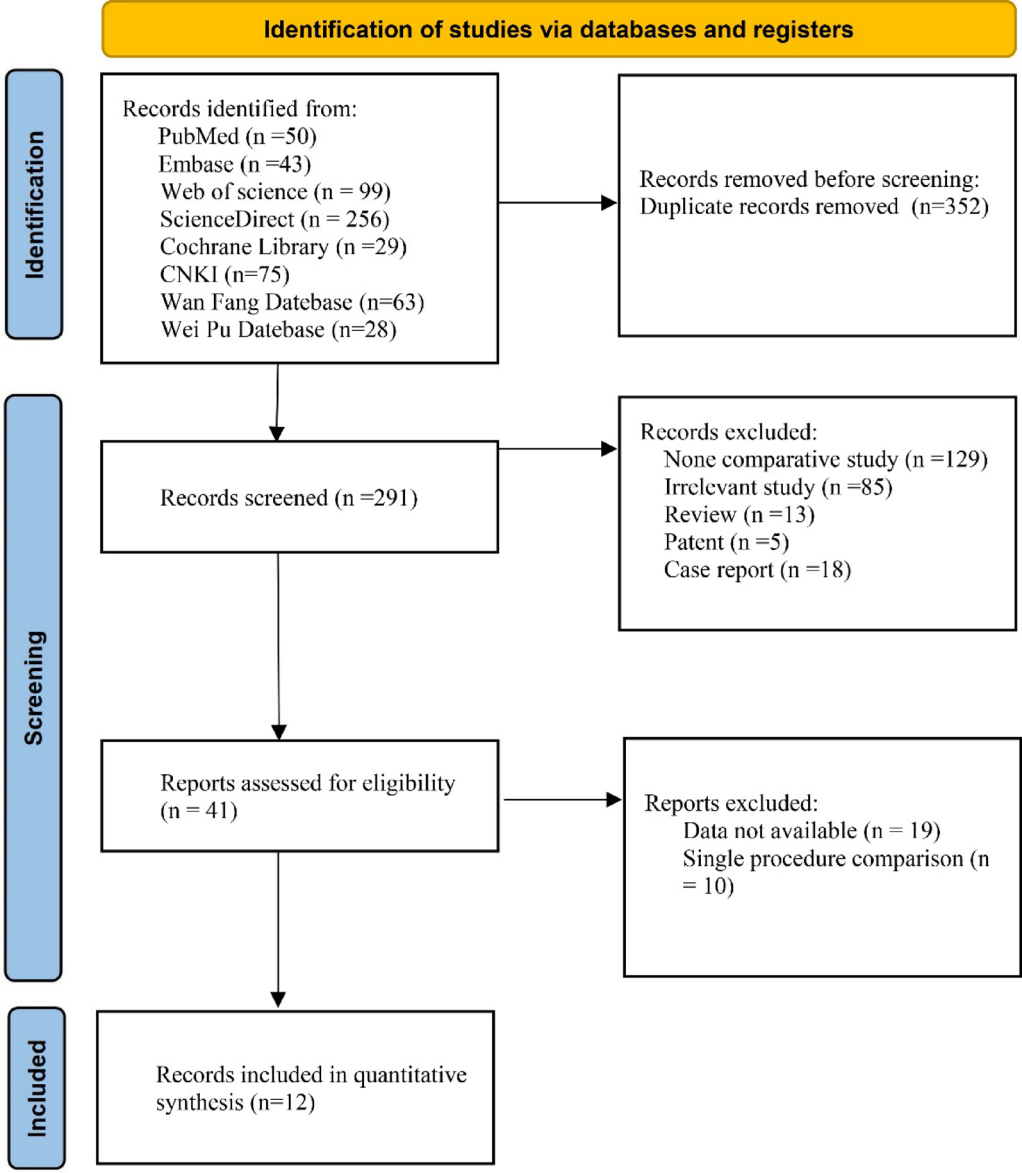
Literature screening flowchart

We conducted a computerized search of PubMed, Embase, ScienceDirect, Web of Science, Cochrane Library, China National Knowledge Infrastructure (CNKI), Wan Fang Database, and Wei Pu Database to gather controlled clinical studies on the efficacy and safety of 3D printed artificial vertebral body in spinal reconstruction after total en bloc spondylectomy (TES) for spinal tumors. The search spanned from the establishment of the databases to February 2024. Language restrictions were applied, including English and Chinese. By preserving the literature that offered the most comprehensive information for overlapping patients, information duplication was avoided. The brief retrieval formula was “((3D printed artificial vertebral body) OR (Three Dimensional printed artificial vertebral body) OR (Titanium Mesh Cage)) AND ((spinal neoplasms) OR (spinal tumors))”.The literature screening process is shown in Fig. 1.

### Inclusion and exclusion criteria

The inclusion criteria were as follows: (1) Study type: randomized controlled trial (RCT) and non-randomized controlled trial (NRCT); (2) Study subjects: Patients with pathological results confirmed as spinal tumors who underwent total spondylectomy and underwent spinal reconstruction using 3D printed artificial vertebral bodies or other implants (such as titanium mesh, titanium cage); (3) Intervention measures: 3D printed artificial vertebral bodies were placed intraoperatively in the 3D group, and titanium mesh and titanium cage were placed intraoperatively in the titanium mesh group; (4) Outcome measures: intraoperative blood loss, operation time, Japanese orthopaedic association (JOA) Scores, Visual analogue scale (VAS), loss of Cobb angle, hospital length stay and one or more of the Frankel classification of spinal cord injury (classification A to E, respectively, were scored 1–5).

Exclusion criteria were as follows: (1) Literature lacking complete information and with unclear or unextractable data; (2) review, meeting, expert opinion, case report, literature that could not obtain the full text; (3) animal experiments, in vitro/biomechanical studies.

## Literature screening and data extraction

Two researchers independently carried out a literature review, meticulously following predefined inclusion and exclusion criteria, and undertook data extraction and mutual verification processes. In cases of discord, they aimed for resolution via thorough discussion. Whenever required, the insight of a third investigator was solicited, and data extraction was executed utilizing a meticulously designed template. The primary data elements extracted encompassed: (1) General details of the included studies, such as title, authorship, and year of publication; (2) Study demographics, including geographical location, sample size, age demographics, duration of operation, and follow-up duration; (3) Clinical outcomes of interest, covering intraoperative blood loss, operation time, Japanese orthopaedic association (JOA) Scores, Visual analogue scale (VAS), Frankel classification, loss of Cobb angle (The segmental angle was measured after surgery and at the last follow-up, that is, the angle formed by the lower endplate of the adjacent upper vertebral body and the upper endplate of the lower vertebral body, and the angle was recorded as positive anteriorly and negative posteriorly, and the amount of angle loss was calculated.), hospital length stay and complications; (4) Critical aspects of bias risk assessment, including the methodology of study population selection, group comparability, and the approaches used for the measurement of exposure variables.

### Literature quality evaluation

The risk of bias assessment for the included studies was independently executed by two evaluators and was followed by a thorough cross-verification process. In instances where there was a disagreement regarding the assessment results, a third evaluator was called upon to mediate discussions and assist in reaching a consensus. The evaluation of bias risk utilized the 5.4 Bias Risk Assessment Tool recommended by the Cochrane Handbook, focusing on elements such as sequence generation, allocation concealment, blinding, integrity of data, selective reporting, and other potential sources of bias. The assessed risk levels were classified as “low risk,” “high risk,” or “unclear risk.” Furthermore, the quality of cohort study literature was appraised using the Newcastle-Ottawa Scale (NOS) criteria, with studies achieving a score of ≥ 7 being deemed as high quality [11].

### Statistical analysis

Meta-analysis of the data from the included articles was performed using RevMan 5.4 software. Continuous variables were expressed as mean difference (MD) and dichotomous variables as odds ratio (OR), and the size of each pooled effect size and its 95% confidence interval (CI) were calculated. Heterogeneity was analyzed using the Chi-square test, and the size of heterogeneity was judged based on the *I*^2^ value. When *P* > 0.1 or *I*^2^ ≤ 50%, heterogeneity between studies was not significant and fixed effect model was used for analysis; if *P* ≤ 0.1 or *I*^2^ > 50%, heterogeneity between studies was significant, and random effect model was used for analysis.

Sensitivity analyses were conducted to evaluate the robustness of the findings. This involved individually removing each study from the pool and conducting a meta-analysis on the remaining studies to see if the overall results held consistent. To investigate the potential for publication bias for each risk factor, we employed Egger’s test, which examines the relationship between the effect sizes and their standard errors. A P value of less than 0.1 in this context suggested a statistically significant difference [12, 13], indicating potential bias. For all conducted statistical tests, a P value of less than 0.05 was deemed to signify statistical significance.

## Results

### Literature screening procedure and results

In this study, 643 papers were obtained through a preliminary search, 352 repeated publications were eliminated by software, titles, and abstracts were read, and 250 papers that obviously did not meet the inclusion criteria were eliminated. After careful reading of the full text and quality evaluation, 29 unqualified papers were further excluded, and 12 qualified papers 14–25] were finally included. The paper screening process is presented in Fig. 1. A total of 489 patients were included, including 234 patients in the 3D printed artificial vertebral body (3D) group and 255 patients in the titanium mesh cages (TMCs) group. The main characteristics of the included studies are presented in Table 1.

### Quality analysis of included studies

This study included a total of 12 studies.The quality of the non-randomized controlled trials was evaluated employing the Newcastle-Ottawa Scale (NOS). All studies included in the analysis achieved scores ranging from 7 to 9 points, signaling high quality. Table 1 offers a consolidated overview of the quality scores attributed to each study.

**Table 1:** Quality assessment using the newcastle-ottawa quality assessment scale for each none randomized controlled trial

Author/year	Selection of the study population	Comparability between groups	Outcome	Total
Cao [14]	4	2	2	8
Hu [15]	4	2	2	8
Chen ZJ [16]	4	3	2	9
Zhang [17]	4	2	2	8
Chen ZY [18]	4	3	2	9
Qing [19]	4	2	2	8
Song [20]	3	2	2	7
Wang L [21]	3	3	2	8
Zhou [22]	3	2	2	7
Ji [23]	3	3	2	8
Li [24]	4	3	2	9
Wang XX [25]	3	2	2	7

### Meta-analysis results

#### Operation time

**Fig. 2:**
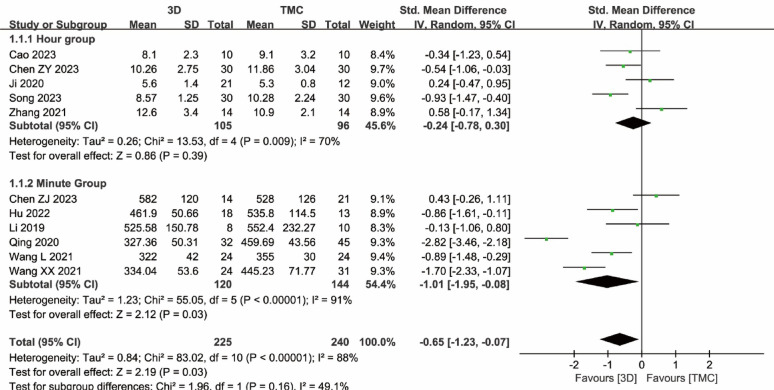
Surgical time

A total of 11 studies used operation time as an outcome measure, with 225 patients in the 3D group and 240 patients in the TMC group. The heterogeneity test (*P* < 0.00001, *I*^2^= 88%), suggested that there was significant heterogeneity between the studies, and a meta-analysis using a random-effects model showed that: [MD = − 0.65, 95% CI (− 1.23, − 0.07), *P* = 0.03] (Fig. 2), The results showed that the operation time was longer in TMC compared to 3D.

### Intraoperative blood loss

**Fig. 3:**
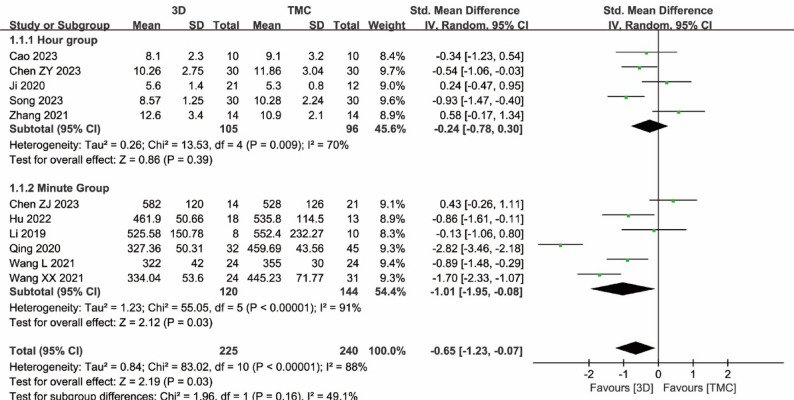
Intraoperative blood loss

Intraoperative blood loss was counted in 11 studies, with 225 patients in the 3D group and 240 patients in the TMC group. The heterogeneity test (*P* < 0.00001, *I*^2^= 94%), suggested that there was significant heterogeneity between the studies. The results showed that intraoperative blood loss in the 3D group was significantly lower than that in the TMC group [MD = − 1.47, 95% CI (− 2.38, − 0.55), *P* = 0.002] (Fig. 3), indicating that 3D printed artificial vertebral body had a certain effect on the reduction of intraoperative blood loss in patients.

### Pain evaluation

Preoperative VAS scores were reported in six studies. The results of the heterogeneity test indicated no significant variance, with *P* = 0.59 and *I*^2^ = 0%. These findings suggest that there was no notable difference in preoperative VAS scores between the 3D and TMC groups [MD = 0.04, 95% CI (− 0.27, 0.35), *P* = 0.82].

**Fig. 4:**
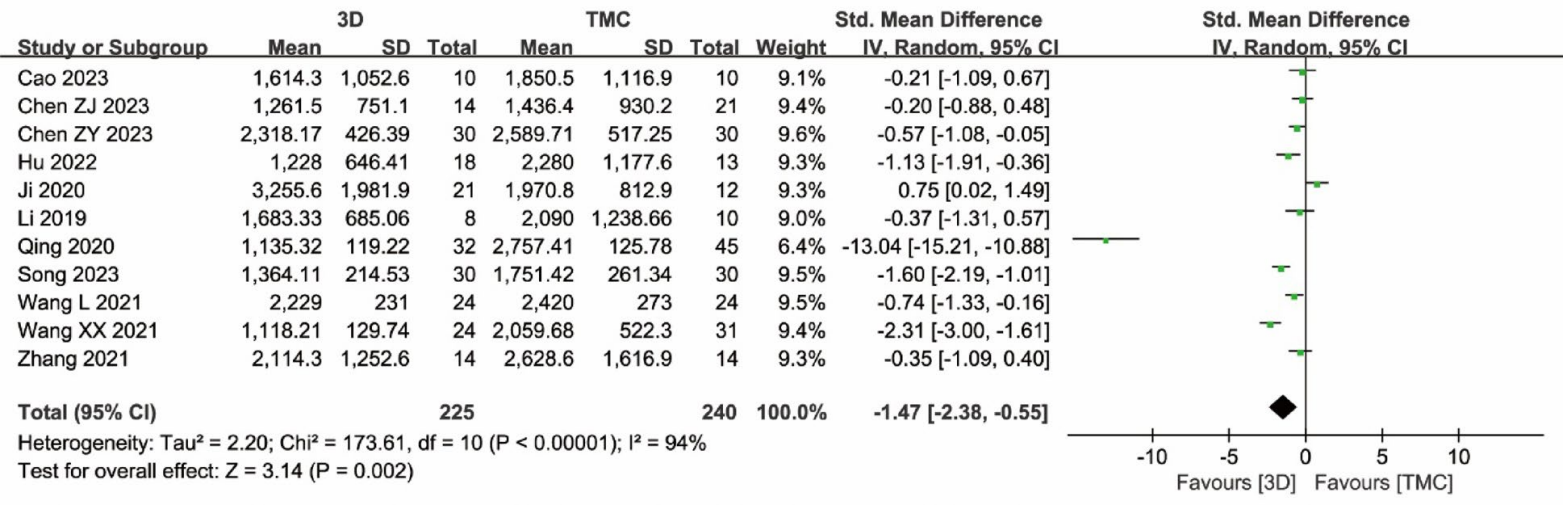
Pain score

VAS scores at early postoperative were reported in 8 papers, and heterogeneity test results showed *P* < 0.0001; *I*^2^= 77%. The results showed that VAS score at early postoperative in 3D group was significantly lower than that in TMC group [MD = − 0.47, 95% CI (− 0.75, − 0.19), *P* = 0.001] (Fig. 4).

### Frankel classification

Preoperative Frankel classification was reported in 3 papers, and heterogeneity test results showed *P* = 0.57; *I*^2^= 0%. The results showed that there was no significant difference in preoperative Frankel classification between 3D and TMC group [MD = − 0.00, 95% CI (− 0.24, 0.23), *P* = 0.97].

**Fig. 5:**
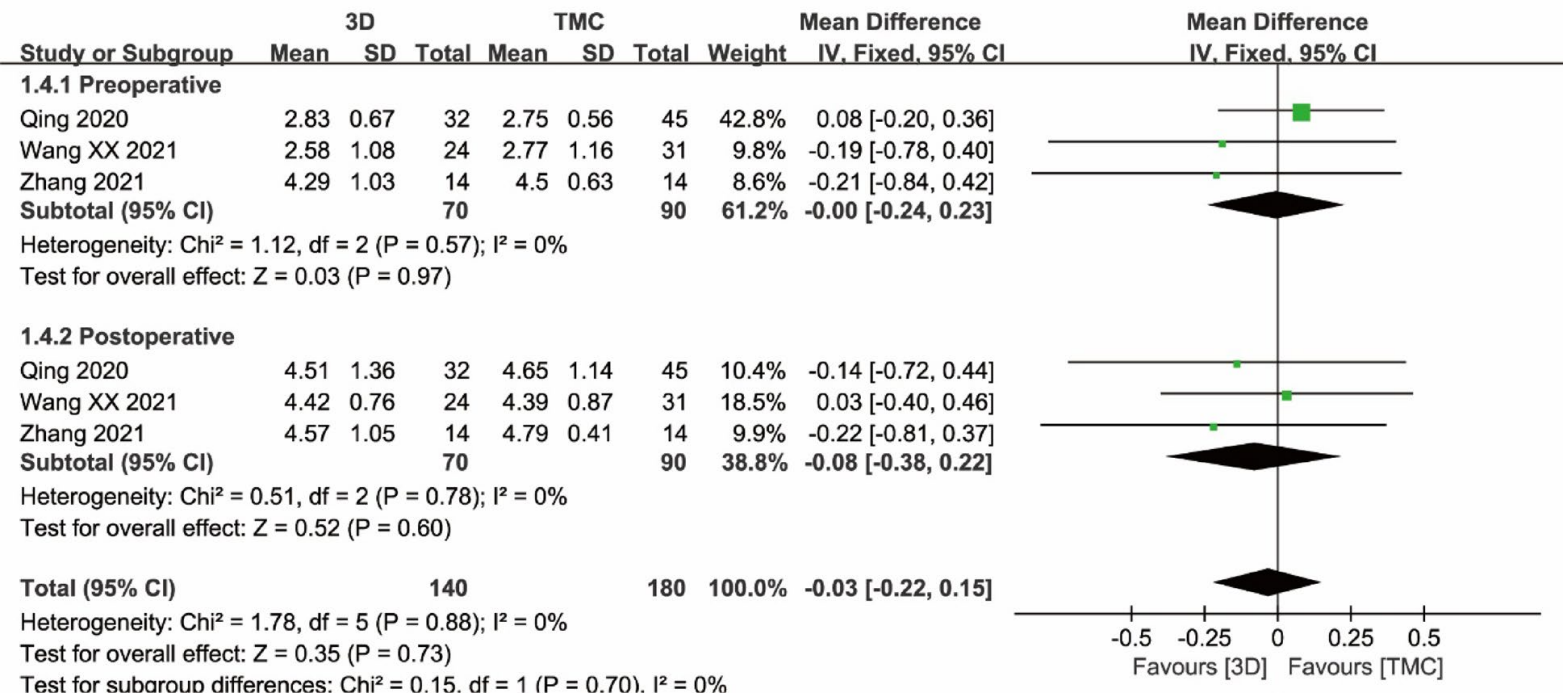
Frankel classification

Postoperative Frankel classification was reported in 3 papers, and heterogeneity test results showed *P* = 0.78; *I*^2^= 0%. The results showed that there was no significant difference in postoperative Frankel classification between 3D and TMC group [MD = − 0.08, 95% CI (− 0.38 0.22), *P* = 0.60] (Fig. 5).

### Neurological function improvement rate

**Fig. 6:**
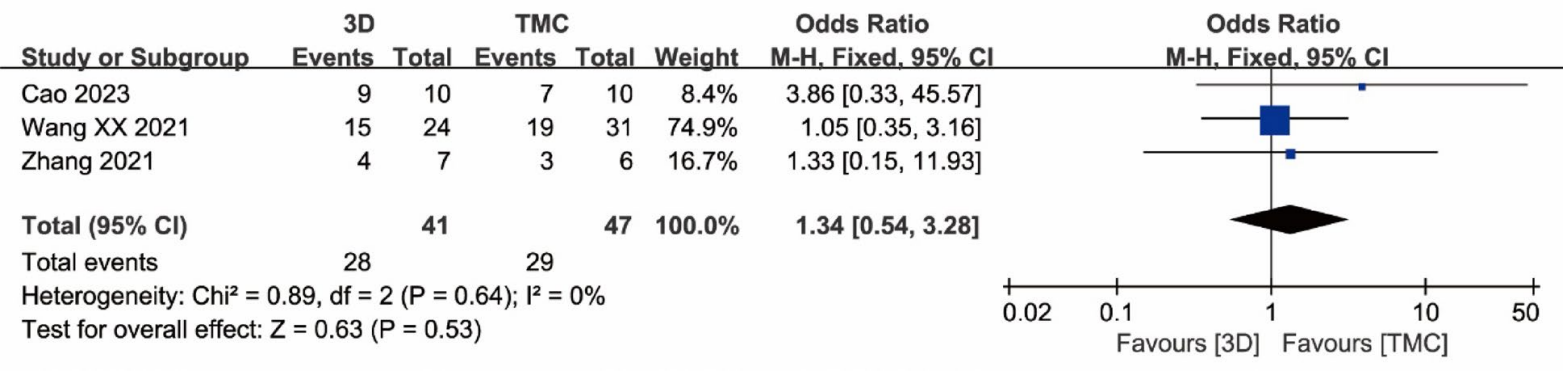
Improvement rate of neurological function

Neurological function improvement rate was reported in 3 papers, heterogeneity test result *P* = 0.64; *I*^2^=0%. There was with no significant heterogeneity among various studies. Meta-analysis of the neurological function improvement rate using the fixed effects model showed that the neurological function improvement rate in the 3D group was slightly lower than that in the TMC group [OR = 1.34, 95% CI (0.54, 3.28), *P* = 0.53, Fig. 6], suggesting that there was no statistically significant difference in neurological function improvement rate between the two groups.

### Japanese orthopaedic association (JOA) Scores

Preoperative Japanese orthopaedic association (JOA) scores was reported in 3 papers, and heterogeneity test results showed *P* = 0.70; *I*^2^= 0%. The results showed that there was no significant difference in the preoperative JOA scores between 3D and TMC group [MD = 0.10, 95% CI (− 0.03, 0.24), *P* = 0.14].

**Fig. 7:**
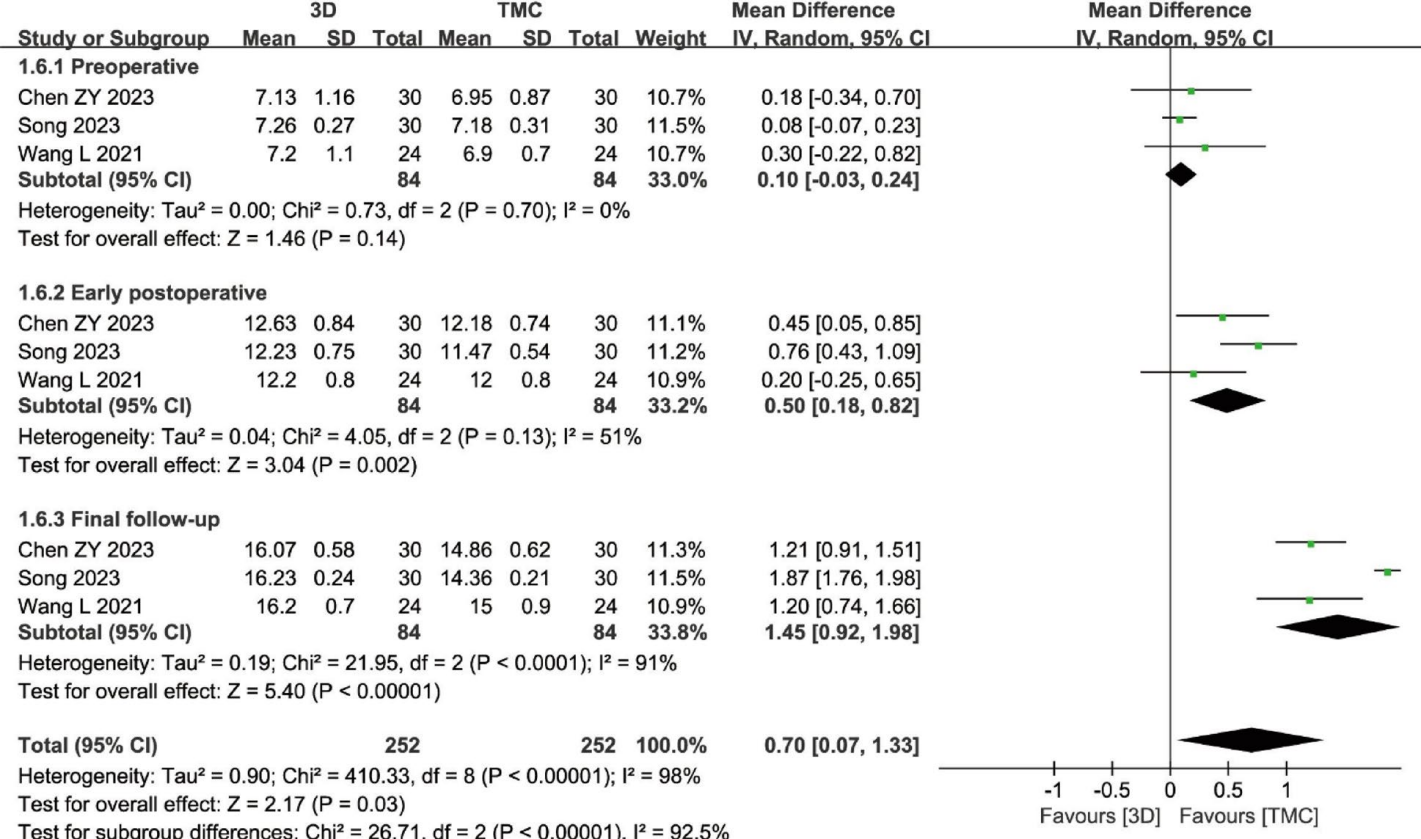
JOA

Postoperative JOA scores was reported in 3 papers, and heterogeneity test results showed *P* = 0.13; *I*^2^= 51%. The results showed that JOA scores at early postoperative in TMC group was significantly lower than that in 3D group [MD = 0.50, 95% CI (0.18, 0.82), *P* = 0.002] (Fig. 7).

### Loss of Cobb angle

**Fig. 8:**
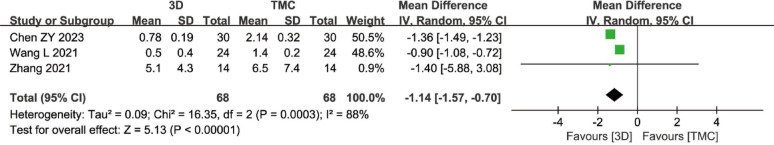
Loss of Cobb angle

Loss of Cobb angle was reported in 3 papers, and heterogeneity test results showed *P* = 0.0003; *I*^2^= 88%. The results showed that loss of Cobb angle in 3D group was significantly lower than that in TMC group [MD = − 1.14, 95% CI (− 1.57, − 0.70), *P* < 0.00001] ( Fig. 8).

### Hospital length stay

**Fig. 9:**
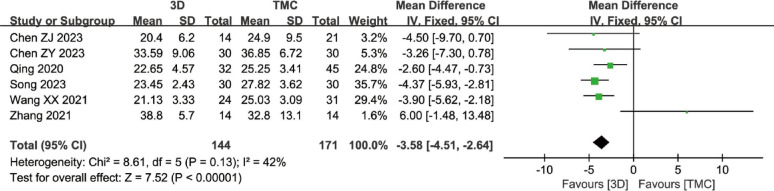
Length of stay

Hospital length stay was reported in 6 papers, heterogeneity test result *P* = 0.13; *I*^2^=42%. There was with no significant heterogeneity among various studies. Meta-analysis of the hospital length stay using the fixed effects model. The results showed that hospital length stay in the 3D group was significantly lower than that in the TMC group [MD = − 3.58, 95% CI (− 4.51, − 2.64), *P* < 0.00001] (Fig. 9), indicating that 3D printed artificial vertebral body had a certain effect on the reduction of hospital length stay in patients.

### Mortality

**Fig. 10:**
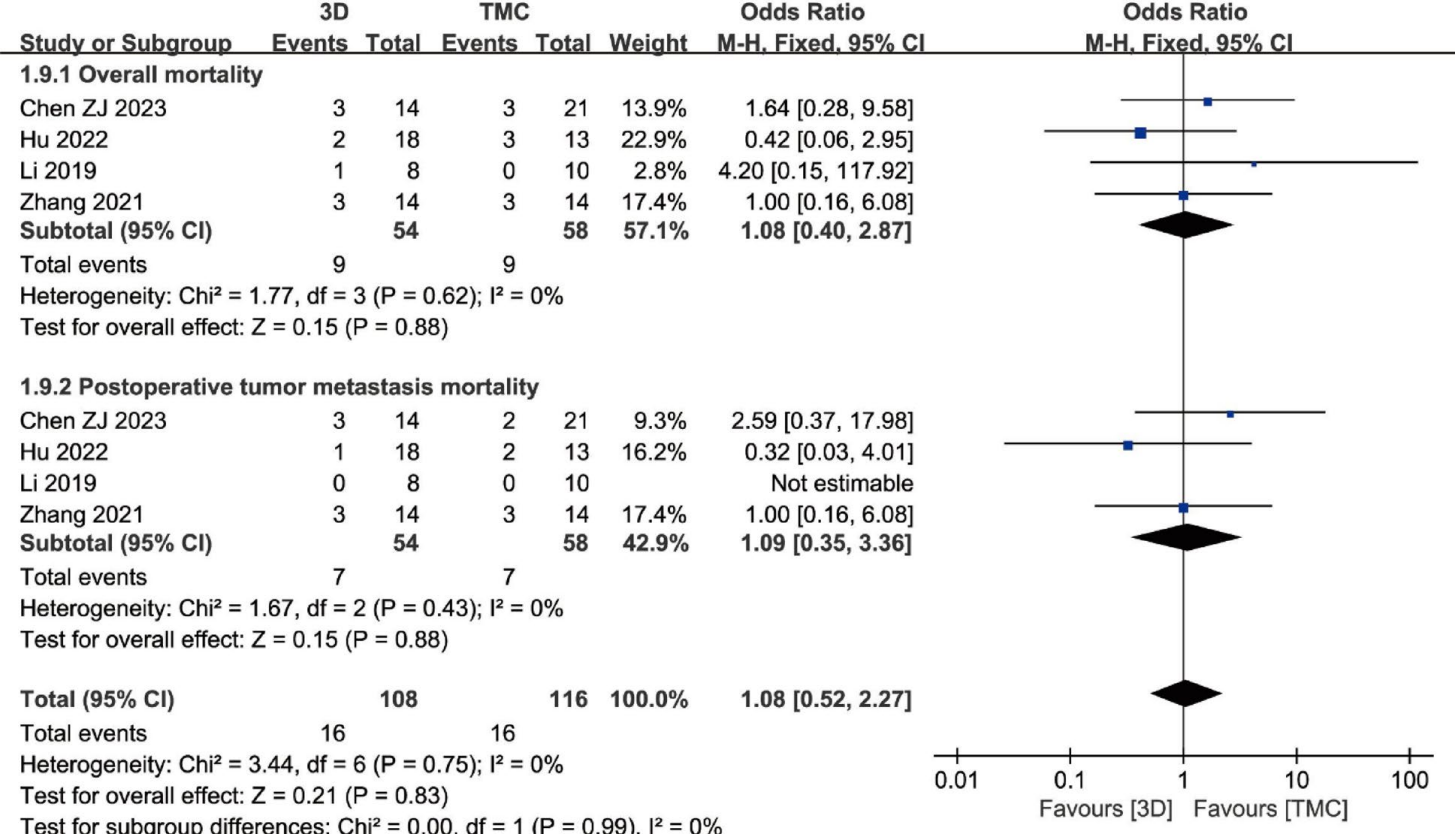
Mortality

Overall mortality was reported in 4 papers, heterogeneity test result *P* = 0.62; *I*^2^=0%. There was with no significant heterogeneity among various studies. Meta-analysis utilizing a fixed effects model revealed that the overall mortality in the 3D group was not significantly different from that in the TMC group [OR = 1.08, 95% CI (0.40, 2.87), *P* = 0.88, Fig. 10].

### Vertebral body subsidence rate

**Fig. 11:**
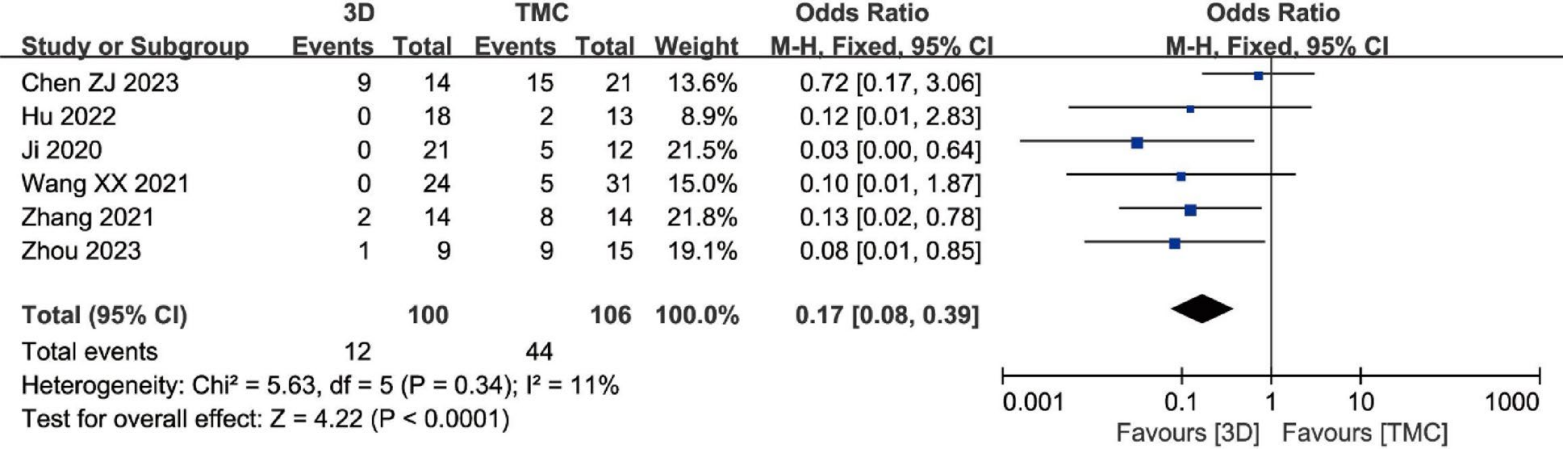
Vertebral sinking rate

Vertebral body subsidence rate was counted in 6 studies, with 100 patients in the 3D group and 106 patients in the TMC group. The heterogeneity test (*P* = 0.34, *I*^2^= 11%). There was with no significant heterogeneity among various studies. The results showed that vertebral body subsidence rate in the 3D group was significantly lower than that in the TMC group [OR = 0.17, 95% CI (0.08, 0.39), *P* < 0.0001] (Fig. 11), indicating that 3D printed artificial vertebral body had a certain effect on the reduction of vertebral body subsidence rate in patients.

### Complications

**Fig. 12:**
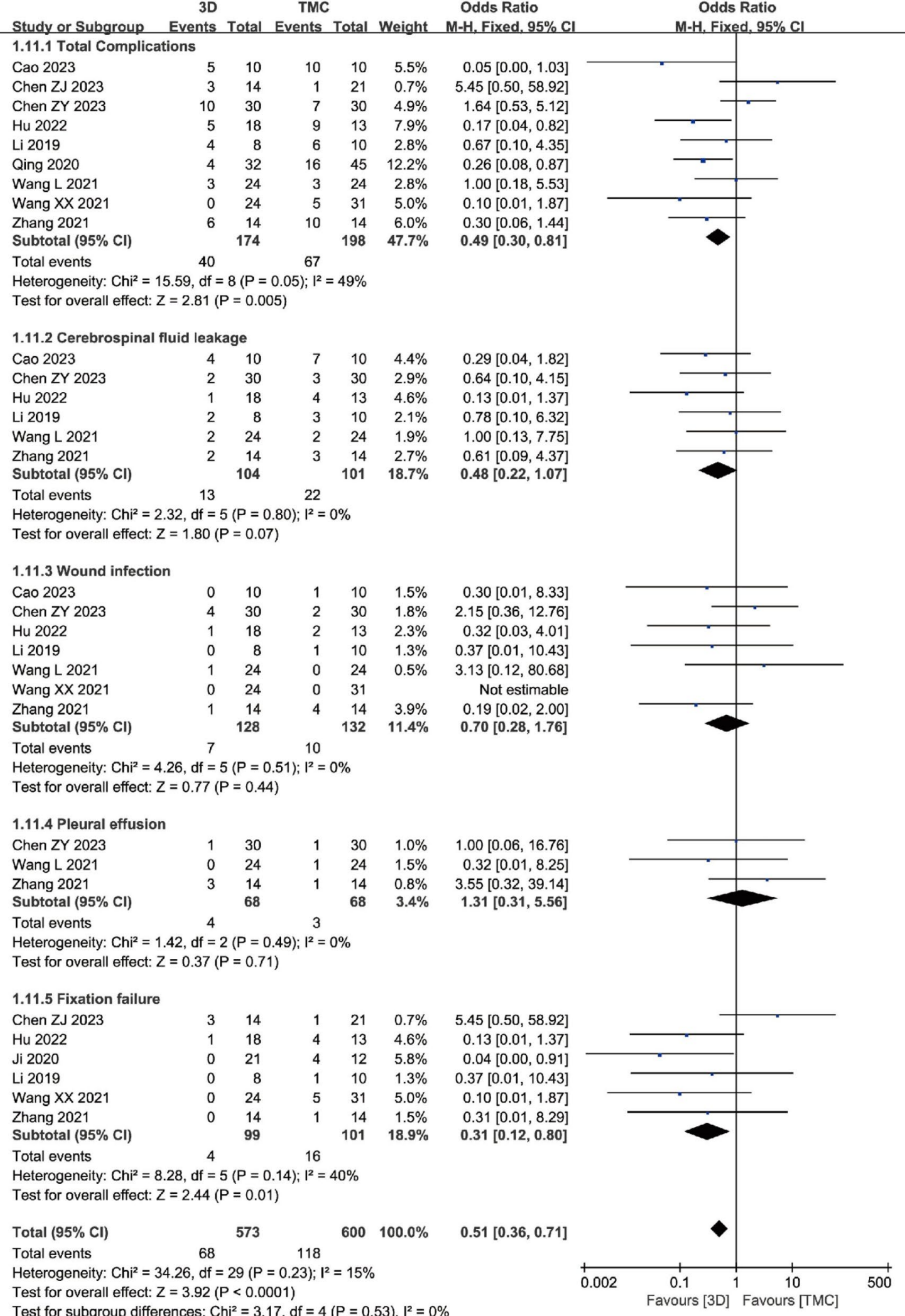
Complication

Total complications were reported in 9 papers, and heterogeneity test results showed *P* = 0.05; *I*^2^= 49%. The results showed that total complications rate in the 3D group was significantly lower than that in the TMC group [OR = 0.49, 95% CI (0.30, 0.81), *P* = 0.005]. Fixation failure were reported in 6 papers, and heterogeneity test results showed *P* = 0.14; *I*^2^= 40%. The results showed that fixation failure rate in the 3D group was significantly lower than that in the TMC group [OR = 0.31, 95% CI (0.12, 0.80), *P* = 0.01] (Fig. 12).

### Heterogeneity and sensitivity analysis

**Fig. 13:**
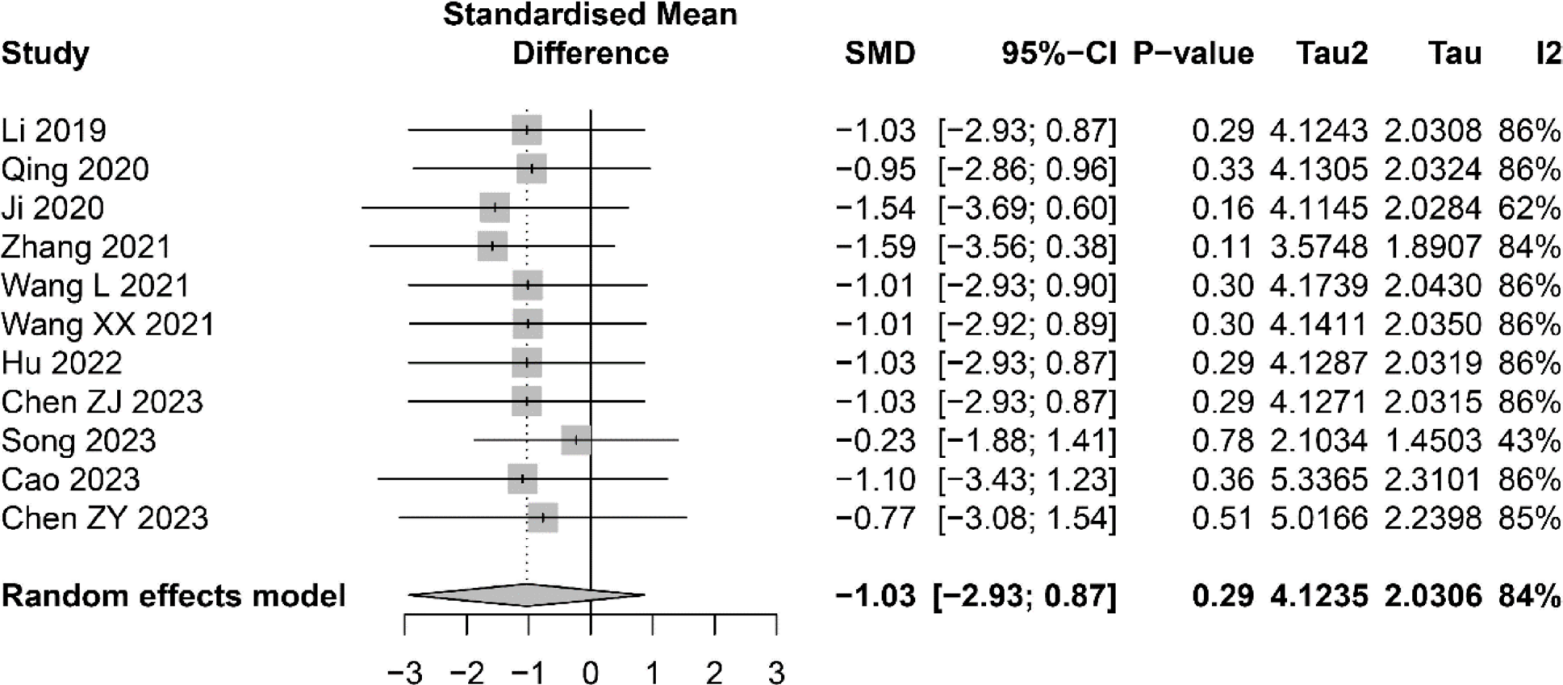
Sensitivity analysis of operative time

The analysis indicated significant variability in operation duration. To evaluate how this variability affected the results, a sensitivity analysis was performed by excluding individual studies from the operation time assessment (Fig. 13). The outcomes of this analysis aligned with the original findings, implying that the heterogeneity had a negligible impact on the overall study conclusions. Potential contributors to this variability include variations in surgeon experience, surgical techniques, methods of measuring intraoperative blood loss, and the comprehensiveness and precision of case documentation.Furthermore, we conducted subgroup analysis for heterogeneity assessment, examining surgical time by ‘surgical segment’ and ‘surgical site.’ These analyses indicated that these two factors were neither the primary nor the sole cause of the overall high heterogeneity. The persistent high heterogeneity suggests the presence of other unmeasured or unanalyzed confounding factors, such as surgical approach, tumor type, surgeon experience, or differences in patient baseline characteristics (See Supplementary Figs. 1, 2).

### Publication deviation

**Fig. 14:**
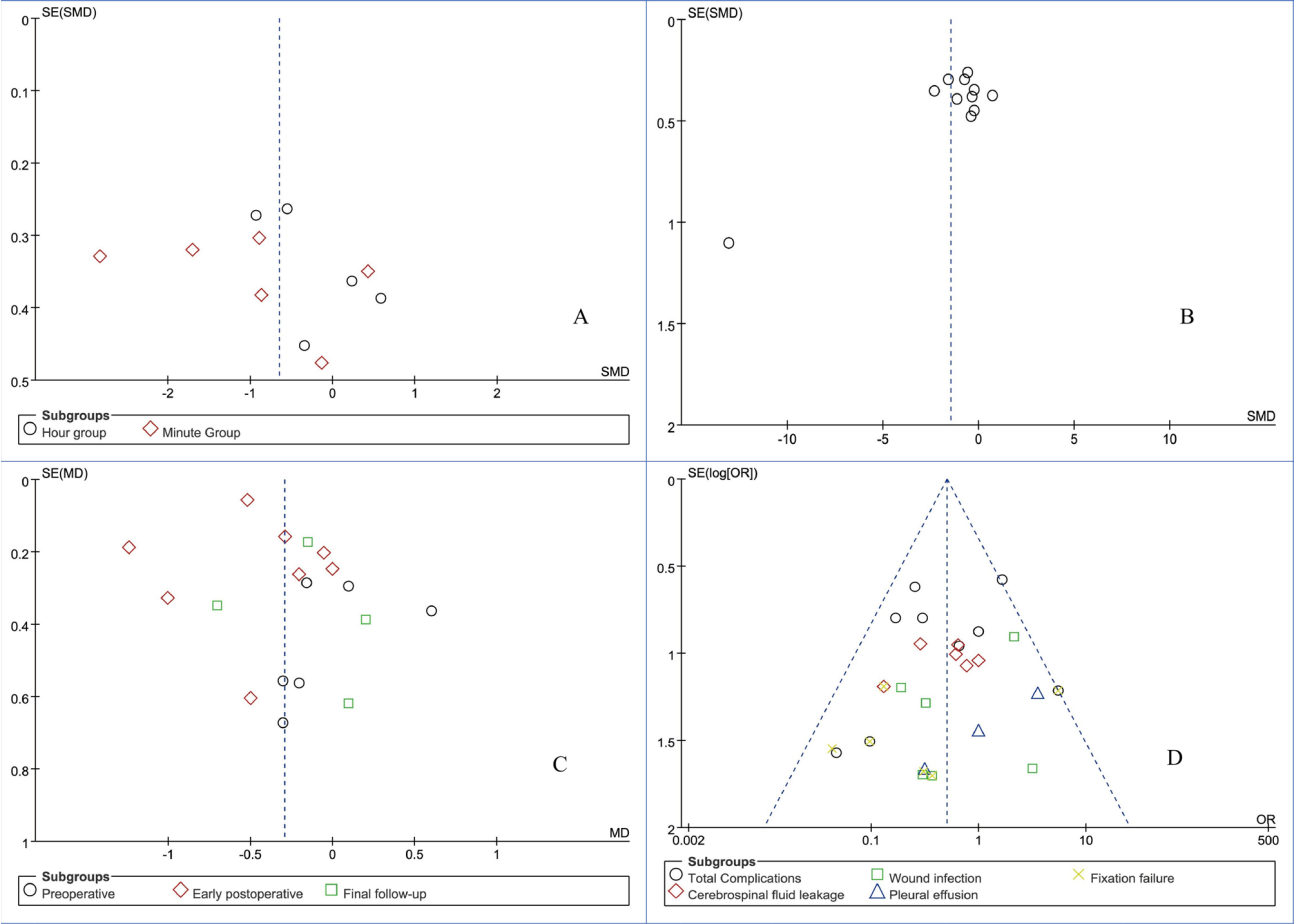
Analysis of publication bias. **A** Surgical duration; **B** intraoperative blood loss; **C **VAS; **D **complications

**Fig. 15:**
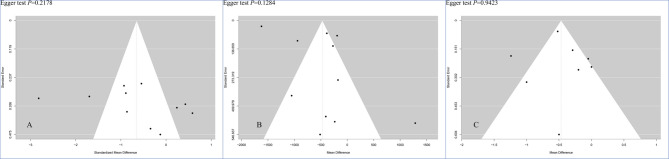
Egger test **A** operative time; **B** intraoperative blood loss; **C** VAS

This study included 12 articles and performed publication bias testing on indicators with more than 10 included studies. Visual assessment of funnel plots for each outcome indicator revealed a generally symmetrical distribution, indicating a low likelihood of publication bias (Fig. 14). Additionally, we conducted bias testing for operative time, intraoperative blood loss, and postoperative VAS. The results of the Egger test are detailed in Fig. 15.

## Discussion

The objectives of Total En Bloc Spondylectomy (TES) spinal reconstruction include: (1) restoring the weight-bearing capacity of the anterior column; (2) filling bone defects; and (3) correcting local spinal deformities caused by tumors [7]. In recent years, the increasingly widespread application of digital technology in orthopedics, particularly the ongoing advancements in 3D printing technology, has enabled the reconstruction of spinal bone defects using customized artificial vertebral bodies [26–28]. In this study, we systematically evaluated the efficacy and safety of spinal reconstruction with 3D printed artificial vertebral bodies for TES in spinal tumors and confirmed that it has advantages in TES spinal reconstruction.

The results of this study showed that the operation time and intraoperative blood loss of TES with 3D printed artificial vertebral body for spinal reconstruction were lower than those of other implants. The author analyzed that it may be related to the following two factors. 3D printed individualized artificial vertebral body can measure the prosthesis with the corresponding height prepared to reduce its error with the real defect height [29], while titanium mesh needs to manually intercept the corresponding length, which may have the error caused by the measurement [30–32]. In addition, there are differences in the processes between the two. The slope or arc design of 3D printing individualized artificial vertebral body upper and lower edges is beneficial to improve its degree of coincidence with adjacent endplates, thereby reducing the difficulty of installation and installation time, thereby reducing the intraoperative blood loss; while the titanium mesh needs to be repeatedly trimmed and check its degree of coincidence with adjacent endplates, so the operation time is longer [23].

In this study, the VAS scores of patients in both groups exhibited a decreasing trend before surgery, 24 h after surgery, and at the last follow-up. The reduction in pain following spinal cord reconstruction with 3D printed artificial vertebral bodies was significantly greater than that observed with titanium cages or titanium mesh. Furthermore, compared to the TMC group, the 3D group showed higher postoperative JOA scores, indicating that reconstruction of spinal sequences with individually tailored 3D printed artificial vertebral bodies is advantageous for enhancing spinal nerve function recovery. The spongy microporous structure of 3D printed individualized artificial vertebral bodies facilitates tissue fluid flow and promotes osteocyte proliferation and migration. Studies have shown that structures with porosities of 70% to 80% are most conducive to bone ingrowth [33, 34]. The 3D printed individualized artificial vertebral body surface has a complex microstructure, which facilitates the accumulation of anti-inflammatory factors, promotes osteoblast differentiation, and also creates a special cellular environment for bone formation [35, 36].

Compared with the TMC group, the 3D group had a smaller decrease in Cobb angle after surgery, suggesting that 3D printing individualized artificial vertebral body reconstruction of spinal sequences is beneficial to reduce the implant subsidence rate. Because the degree of fit between the endplate and the titanium mesh is limited, the contact surface between the titanium mesh and the endplate is small, the spinal alignment may deviate from the contact surface, and uneven stress may occur, resulting in excessive local pressure, resulting in the inversion effect of the implant, forming an included angle, which is not conducive to bone fusion, resulting in height loss and prolonging the healing time [17, 20]. In contrast, endplates and 3D printed individualized artificial vertebral bodies have a larger contact area and a higher degree of conformity, and pressure is transmitted through the vertebral body in the form of surface and surface contact, which disperses weight-bearing strength and relieves collapse.

Tumor resection, spinal cord decompression and stability reconstruction are the basic elements of en bloc total spondylectomy. Because the whole vertebral segment is removed together with ligaments and part of muscle tissue, the anterior, middle and posterior column structures are completely destroyed, so stability reconstruction must be performed [37]. The goal of reconstruction is to achieve long-term biological fusion. Although titanium cages play an effective supporting role, the long-term reliability has been gradually questioned. Chen et al. [38] followed 300 cervical cage reconstruction cases and found cage subsidence to be common and associated with postoperative complications. Yoshioka et al. [39] found that internal fixation failure is highly likely to occur after long-segment thoracolumbar total spondylectomy and intervertebral space collapse due to titanium cage subsidence, segmental angle is difficult to maintain, and biostability is lost, resulting in screw and rod stress concentration and a greatly increased risk of breakage. This study showed that the 3D printed artificial vertebral body had a lower subsidence rate compared with other implants, and the reasons were analyzed: (1) Metal implants such as titanium cage titanium mesh usually did not have an arc, and the physiological curvature of the spine made the implants often have a certain angle with the horizontal plane, the spinal alignment easily deviated from the bone contact surface, and the stress was unevenly distributed in the titanium mesh or titanium cage. (2), the elastic modulus of metal implants did not match significantly with adjacent vertebral bodies and were higher than bone [40]. Because of its sharp edge, small contact area and high elastic modulus, it produces stress shielding effect and causes bone tissue loss from adjacent vertebral bodies, resulting in osteoporosis. (3), often resulting in displacement of the implant at an angle to the endplate of the adjacent vertebral body under the continuous action of stress concentration and inhomogeneity. Occurrence of fracture collapse of the titanium mesh or cage, or fracture of the adjacent vertebral body due to excessive stress and its sharp edges, directly destabilizes the spine and increases the risk of implant subsidence [23]. 3D printed artificial vertebral body curvature is designed according to the location and physiological curvature of the tumor, with a large contact area and better conformity of the adjacent vertebral body surface. The stress distribution load is loaded to the periphery of the endplate with thick cortical bone, the spinal alignment is not easy to deflect, the stress distribution is relatively more uniform and always conducts through the intervertebral contact surface, and its three-dimensional microporous structure makes it have high strength and low modulus. It is not easy to occur stress shielding effect, has greater compressive resistance, is not easy to collapse and deform, provides better stability, and reduces the settlement rate of implants.

Although en bloc resection of spinal tumors can significantly improve the prognosis of patients, surgery-related complications are not uncommon and sometimes fatal. Boriani et al. [41] retrospectively reviewed the medical records of 134 patients (90 primary tumors and 44 metastatic tumors) who underwent en bloc resection and found that 47 (34.3%) patients developed complications, 70 complications, including 41 severe and 29 mild complications, and 3 (2.0%) patients died of complications. Risk factors causing complications include previous irregular surgery, multilevel resection, and one-stage combined approach surgery. Among procedure-related complications, fatal complications require more vigilance and attention from physicians [24]. The perioperative mortality of en bloc resection of primary spinal tumors ranges from 0 to 7.7%, respiratory failure is the most common cause of death, and wound complications and massive hemorrhage are the most common complications. Intraoperative macrovascular injury is often the most dangerous complication [42]. In this study, there was no statistically significant difference in overall mortality between the two groups, with only 5.4% in 3D group versus 7.5% in TMC group regarding postoperative wound infections. For patients with spinal tumors, postoperative infection is often catastrophic, and it will also delay the subsequent radiotherapy, chemotherapy, etc., so it is recommended to actively take preventive antibiotic application regimen. Internal fixation failure is one of the common late surgical complications, especially with advances in oncological treatment outcomes, patients achieve longer survival and put forward higher requirements for the reliability of spinal reconstruction. In this study, the instrumentation failure rate was only 4% in the 3D group and 15.8% in the TMC group, en bloc total vertebrectomy necessitates the removal of the vertebral body, disc, and all adnexal structures, leading to significantly unstable mechanical structures. Strong cephalocaudal pedicle screw fixation is essential, along with the reconstruction of anterior column support function, to maintain spinal stability. To ensure long-term stability of the entire support system, it is typical to employ titanium mesh, bone cement, and other supports anteriorly, complemented by the use of autologous or allogeneic bone for biological fusion with adjacent normal vertebrae. However, nonfusion of the bone graft often occurs due to the requirement for radiotherapy and chemotherapy in cancer patients, or due to compromised physical condition; additionally, subsidence of the titanium mesh can result from factors such as the small diameter of the anterior titanium mesh or the cutting of the endplate [43]. These issues frequently lead to the failure of the entire fixation system, causing fractures or displacement of the hardware. Sciubba et al. [44] reported 9 instrumentation failures after 23 (39.1%) total vertebrectomy procedures. Park et al. [45] reported rod breakage in 12 of 32 (37.5%) cases following total or sagittal vertebrectomy and their team found that anterior instrumentation with structural bone blocks filled with autologous or decalcified bone matrix failed less frequently (16.7%) than with titanium mesh (46.2%) and telescopic titanium mesh (38.5%), suggesting that a large endplate contact area contributes to strong bony fusion. 3D printed artificial vertebral bodies have a larger contact area than titanium mesh and structural bone graft blocks, providing more reliable support.

This study has the following limitations: All included studies were retrospective, some studies had small sample size, limited data acquisition, and possible selection bias; because 3D printed artificial vertebral bodies were not applied to TES for a long time, The majority of literature included in this study had relatively short follow-up periods (mean/median follow-up duration), primarily providing evidence on the short-to medium-term efficacy and safety of 3D-printed artificial vertebrae. Consequently, their long-term biomechanical durability, final state of bone integration, incidence of late complications, and long-term impact on patients’ quality of life still require confirmation through extended follow-up. To comprehensively validate the long-term advantages of 3D-printed artificial vertebrae in spinal tumor reconstruction, future studies urgently need to conduct rigorously designed, large-sample prospective randomized controlled trials (RCTs) with systematic follow-up periods of up to 5 or even 10 years.The persistent high heterogeneity observed in our subgroup analyses suggests that factors such as surgical approach, tumor type, surgeon experience, or patient baseline characteristics may have contributed to the variability in outcomes. This unexplained heterogeneity may affect the robustness of our conclusions, and future studies should aim to control for these potential confounders through more rigorous study designs, such as prospective randomized controlled trials with standardized protocols.

## Conclusion

The 3D printing group demonstrated advantages in early postoperative pain relief (VAS) and spinal sequence maintenance (loss of Cobb angle), while there were no statistically significant differences between the two groups in Frankel grading and neurological improvement rates. Notably, the traditional titanium mesh group showed more significant improvements in early postoperative JOA scores.

## Supplementary Information

Below is the link to the electronic supplementary material.

Supplementary Material 1

Supplementary Material 2

## Data Availability

The data sets generated and analyzed during the current study are not publicly available but can be obtained from the corresponding author on reasonable request.

## References

[1] Sciubba DM, Goodwin CR, Yurter A, et al. A systematic review of clinical outcomes and prognostic factors for patients undergoing surgery for spinal metastases secondary to breast cancer. Global Spine J. 2016;6(5):482–96. 27433433 10.1055/s-0035-1564807 PMC4947406

[2] Richardson ED, Price DK, Figg WD. Significant addition to treatment options for bone metastasis in prostate cancer. Cancer Biol Ther. 2012;13(2):69–70. 22336908 10.4161/cbt.13.2.18441 PMC3342941

[3] Constans JP, de Divitiis E, Donzelli R, et al. Spinal metastases with neurological manifestations. Review of 600 cases. J Neurosurg. 1983;59(1):111–8. 6864265 10.3171/jns.1983.59.1.0111

[4] Boriani S, Biagini R, De Iure F, et al. En bloc resections of bone tumors of the thoracolumbar spine. A preliminary report on 29 patients. Spine (Phila Pa 1976). 1996;21(16):1927–31. 8875727 10.1097/00007632-199608150-00020

[5] Chi JH, Sciubba DM, Rhines LD, Gokaslan ZL. Surgery for primary vertebral tumors: en bloc versus intralesional resection. Neurosurg Clin N Am. 2008;19(1):111–7. 18156053 10.1016/j.nec.2007.10.004

[6] Enneking WF, Spanier SS, Goodman MA. A system for the surgical staging of musculoskeletal sarcoma. Clin Orthop Relat Res. 1980;2003(415):4–18. 10.1097/01.blo.0000093891.12372.0f 14612624

[7] Girolami M, Boriani S, Bandiera S, et al. Biomimetic 3D-printed custom-made prosthesis for anterior column reconstruction in the thoracolumbar spine: a tailored option following en bloc resection for spinal tumors: Preliminary results on a case-series of 13 patients. Eur Spine J. 2018;27(12):3073–83. 30039254 10.1007/s00586-018-5708-8

[8] Xu N, Wei F, Liu X, et al. Reconstruction of the upper cervical spine using a personalized 3D-printed vertebral body in an adolescent with Ewing Sarcoma. Spine (Phila Pa 1976). 2016;41(1):E50–4. 26335676 10.1097/BRS.0000000000001179

[9] Moher D, Liberati A, Tetzlaff J, Altman DG. Preferred reporting items for systematic reviews and meta-analyses: the PRISMA statement. Int J Surg. 2010;8(5):336–41. 20171303 10.1016/j.ijsu.2010.02.007

[10] Phan K, Mobbs RJ. Systematic reviews and meta-analyses in spine surgery, neurosurgery and orthopedics: guidelines for the surgeon scientist. J Spine Surg. 2015;1(1):19–27. 27683675 10.3978/j.issn.2414-469X.2015.06.01 PMC5039866

[11] Luo M, Cao Q, Wang D, et al. The impact of diabetes on postoperative outcomes following spine surgery: a meta-analysis of 40 cohort studies with 2.9 million participants. Int J Surg. 2022;104:106789. 35918006 10.1016/j.ijsu.2022.106789

[12] Luo M, Cao Q, Zhao Z, et al. Risk factors of epidural hematoma in patients undergoing spinal surgery: a meta-analysis of 29 cohort studies. Int J Surg. 2023;109(10):3147–58. 37318854 10.1097/JS9.0000000000000538 PMC10583939

[13] Peters JL, Sutton AJ, Jones DR, Abrams KR, Rushton L. Comparison of two methods to detect publication bias in meta-analysis. JAMA. 2006;295(6):676–80. 16467236 10.1001/jama.295.6.676

[14] Cao Y, Yang N, Wang S, et al. The application of 3D-printed auto-stable artificial vertebral body in en bloc resection and reconstruction of thoracolumbar metastases. J Orthop Surg Res. 2023;18(1):638. 37644570 10.1186/s13018-023-04135-3 PMC10463335

[15] Hu P, Du S, Wei F, et al. Reconstruction after resection of C2 vertebral tumors: a comparative study of 3D-printed vertebral body versus titanium mesh. Front Oncol. 2022;12:1065303. 36601475 10.3389/fonc.2022.1065303 PMC9806260

[16] Chen Z, Lü G, Wang X, et al. Is 3D-printed prosthesis stable and economic enough for anterior spinal column reconstruction after spinal tumor resection? A retrospective comparative study between 3D-printed off-the-shelf prosthesis and titanium mesh cage. Eur Spine J. 2023;32(1):261–70. 36477893 10.1007/s00586-022-07480-9

[17] Zhang Y, Sun Y, Xiong W, et al. Application of 3D-printed individual artificial vertebral body in reconstruction of thoracolumbar tumor after tobal en-bloc spondylectomy. Orthop Biomech Mater Clin Study. 2021;18(1):17–2126.

[18] Chen Z, Guan K, Sun Y, et al. Clinical value of 3D printed personalized artificial vertebral weight construction for spinal tumor surgery. Guangdong Med J. 2023;44(01):96–101.

[19] Qing P, Cao Y, Xu J, Deng H. Effectiveness analysis of using 3D printing technique in spinal reconstruction post excision of spinal tumors. China Med Equip. 2020;17(1):109–12.

[20] Song R, Cai Y, Zhao Y, Guo H, Wang W. Clinical effect of 3D printed artificial vertebral body for spinal tumor resection and reconstruction. Chin J Experimental Surg. 2023;40(9):1863–5.

[21] Wang L, Gao S, Liu J, et al. Effect of 3D printing artificial vertebral body in reconstruction of spinal stability after total resection of spi-nal metastases. J Practical Med. 2021;37(23):3008–13.

[22] Zhou H, Wang R, Liu Z, et al. 3D-printed vertebral body in anterior spinal reconstruction after total spondylectomy for patients with cervical chordoma. J Peking University(Health Sciences). 2023;55(1):144–8. 10.19723/j.issn.1671-167X.2023.01.022 PMC9894787 36718703

[23] Ji J, Hu Y, Miao J. Application of 3D printed porous artificial vertebra in reconstruction of thoracolumbar tumor. Chin J Orthop. 2020;40(4):208–16.

[24] Li Z, Wei F, Liu Z, et al. Safety and mid-term follow-up results of en bloc resection for primary and metastatic spine tumors based on Weinstein-Boriani-Biagini classification. J Clin Orthop Res. 2019;4(5):261–267280.

[25] Wang X, Ji Y, Leng Z, et al. New 3D printed individualized artificial vertebral body for spinal reconstruction after spinal tumor resection. Chin J Experimental Surg. 2021;38(6):1155–8.

[26] Malik HH, Darwood AR, Shaunak S, et al. Three-dimensional printing in surgery: a review of current surgical applications. J Surg Res. 2015;199(2):512–22. 26255224 10.1016/j.jss.2015.06.051

[27] Martelli N, Serrano C, van den Brink H, et al. Advantages and disadvantages of 3-dimensional printing in surgery: a systematic review. Surgery. 2016;159(6):1485–500. 26832986 10.1016/j.surg.2015.12.017

[28] Xiao JR, Huang WD, Yang XH, et al. En Bloc resection of primary malignant bone tumor in the cervical spine based on 3-dimensional printing technology. Orthop Surg. 2016;8(2):171–8. 27384725 10.1111/os.12234 PMC6584397

[29] Shi L, Li X, Li X, et al. Preliminary study on the a novel individualized 3D printing artificial vertebral body in spine reconstruction. Chin J Orthop. 2020;40(6):335–43.

[30] Wei F, Li Z, Liu Z, et al. Upper cervical spine reconstruction using customized 3D-printed vertebral body in 9 patients with primary tumors involving C2. Ann Transl Med. 2020;8(6):332–332. 32355776 10.21037/atm.2020.03.32 PMC7186708

[31] Tang X, Yang Y, Zang J, et al. Preliminary results of a 3D-printed modular vertebral prosthesis for anterior column reconstruction after multilevel thoracolumbar total En Bloc spondylectomy. Orthop Surg. 2021;13(3):949–57. 33817999 10.1111/os.12975 PMC8126945

[32] Sun Z, Yin M, Sun Y, et al. Customized multilevel 3D printing implant for reconstructing spine tumor: a retrospective case series study in a single center. Orthop Surg. 2022;14(9):2016–22. 35894154 10.1111/os.13357 PMC9483039

[33] Wu SH, Li Y, Zhang YQ, et al. Porous titanium-6 aluminum-4 vanadium cage has better osseointegration and less micromotion than a poly-ether-ether-ketone cage in sheep vertebral fusion. Artif Organs. 2013;37(12):E191–201. 24147953 10.1111/aor.12153

[34] Wang X, Zhai D, Yao X, et al. 3D printing of pink bioceramic scaffolds for bone tumor tissue therapy. Appl Mater Today. 2022;27(3):124–43.

[35] Olivares-Navarrete R, Hyzy SL, Slosar PJ, et al. Implant materials generate different peri-implant inflammatory factors: poly-ether-ether-ketone promotes fibrosis and microtextured titanium promotes osteogenic factors. Spine (Phila Pa 1976). 2015;40(6):399–404. 25584952 10.1097/BRS.0000000000000778 PMC4363266

[36] Olivares-Navarrete R, Hyzy SL, Gittens RS, et al. Rough titanium alloys regulate osteoblast production of angiogenic factors. Spine J. 2013;13(11):1563–70. 23684238 10.1016/j.spinee.2013.03.047 PMC3785549

[37] Kawahara N, Tomita K, Murakami H, Demura S. Total en bloc spondylectomy for spinal tumors: surgical techniques and related basic background. Orthop Clin North Am. 2009;40(1):47–63. vi. 19064055 10.1016/j.ocl.2008.09.004

[38] Chen Y, Chen D, Guo Y, et al. Subsidence of titanium mesh cage: a study based on 300 cases. J Spinal Disord Tech. 2008;21(7):489–92. 18836360 10.1097/BSD.0b013e318158de22

[39] Yoshioka K, Murakami H, Demura S, et al. Risk factors of instrumentation failure after multilevel total en bloc spondylectomy. Spine Surg Relat Res. 2017;1(1):31–9. 31440610 10.22603/ssrr.1.2016-0005 PMC6698537

[40] Ji C, Yu S, Yan N, et al. Risk factors for subsidence of titanium mesh cage following single-level anterior cervical corpectomy and fusion. BMC Musculoskelet Disord. 2020;21(1):32. 31937288 10.1186/s12891-019-3036-8 PMC6961320

[41] Boriani S, Bandiera S, Colangeli S, Ghermandi R, Gasbarrini A. En bloc resection of primary tumors of the thoracic spine: indications, planning, morbidity. Neurol Res. 2014;36(6):566–76. 24725289 10.1179/1743132814Y.0000000369

[42] Yamazaki T, McLoughlin GS, Patel S, Rhines LD, Fourney DR. Feasibility and safety of en bloc resection for primary spine tumors: a systematic review by the Spine Oncology Study Group. Spine (Phila Pa 1976). 2009;34(22 Suppl):S31–8. 19829275 10.1097/BRS.0b013e3181b8b796

[43] Wei F, Liu S, Liu Z, et al. Study on the safety and effectiveness of 3D printed artificial vertebral body reconstruction after en bloc resection of thoracolumbar tumor. Chin J Spine Spinal Cord. 2020;30(9):774–81.

[44] Sciubba DM, De la Garza RR, Goodwin CR, et al. Total en bloc spondylectomy for locally aggressive and primary malignant tumors of the lumbar spine. Eur Spine J. 2016;25(12):4080–7. 27262560 10.1007/s00586-016-4641-y

[45] Park SJ, Lee CS, Chang BS, et al. Rod fracture and related factors after total en bloc spondylectomy. Spine J. 2019;19(10):1613–9. 31059817 10.1016/j.spinee.2019.04.018

