# Supplementary figures and images for: Efficacy and safety of 3D-printed artificial vertebral bodies for spinal tumor resection and reconstruction: a systematic review and meta-analysis
Source: J Orthop Surg Res. 2026 Apr 29;21:384. doi: 10.1186/s13018-026-06788-2 (PMC13359806; doi:10.1186/s13018-026-06788-2)

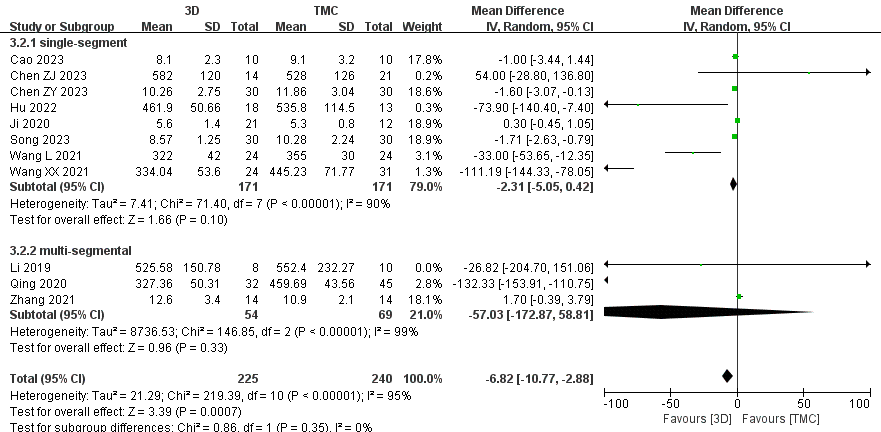

Supplement: Supplementary file 1 — Supplementary Material 1 [file 13018_2026_6788_MOESM1_ESM.png]

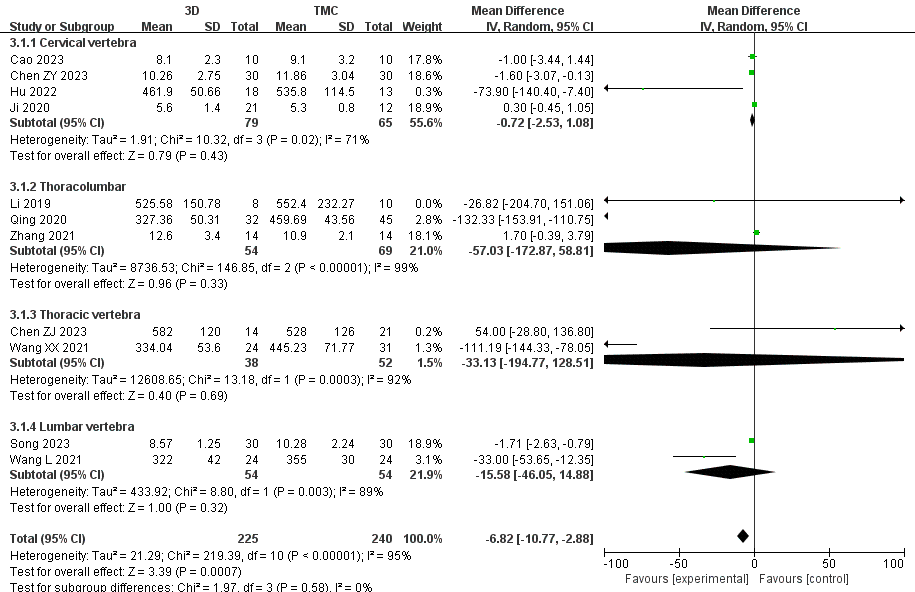

Supplement: Supplementary file 2 — Supplementary Material 2 [file 13018_2026_6788_MOESM2_ESM.png]
